# Epidemiologie des nummulären Ekzems – methodische Ansätze und Ergebnisse aus bundesweiten Routinedaten

**DOI:** 10.1111/ddg.15932_g

**Published:** 2026-07-07

**Authors:** Kristina Hagenström, Katharina Müller, Theresa Klinger, Charlotte Willers, Kilian Eyerich, Matthias Augustin

**Affiliations:** ^1^ Competenzzentrum Versorgungsforschung in der Dermatologie (CVderm) Institut für Versorgungsforschung in der Dermatologie und bei Pflegeberufen (IVDP) Universitätsklinikum Hamburg‐Eppendorf (UKE), Hamburg; ^2^ Abteilung für Dermatologie und Venerologie Universitätsklinikum Freiburg

**Keywords:** Atopische Dermatitis, Daten der gesetzlichen Krankenversicherung, Inzidenz, Krankheitshäufigkeit, Prävalenz, Schweregrad, Validierung, Atopic dermatitis, frequency of illness, incidence, prevalence, severity, statutory health insurance data, validation

## Abstract

**Hintergrund und Zielsetzung:**

Das nummuläre Ekzem (NE) ist eine chronische Hauterkrankung, für die nur wenige epidemiologische Daten vorliegen. Diese Studie schätzt die Prävalenz, Inzidenz und Schwere der Erkrankung in Deutschland.

**Methoden:**

In einer Querschnittsanalyse (2016–2022) wurden anhand von Daten der gesetzlichen Krankenversicherung Personen mit NE (ICD‐10 L30.0) identifiziert und der Schweregrad (Krankenhausaufenthalt, Arbeitsunfähigkeit, systemische Behandlung) sowie Begleiterkrankungen der Haut bewertet.

**Ergebnisse:**

Im Jahr 2022 lag die Prävalenz von NE zwischen 0,07% und 0,26%. Die Inzidenz betrug 0,16%–0,17%. Das Durchschnittsalter lag bei 47,5 Jahren, 57,4% der Patienten waren männlich. Ältere Männer wiesen eine höhere Prävalenz auf, während Kinder unter 6 Jahren (0,34%), insbesondere unter zwei Jahren (0,56%–0,57%), stärker betroffen waren. Schwere NE wurden bei 14,8% der Patienten festgestellt. Atopische Dermatitis trat bei 18% gleichzeitig auf. Personen mit NE hatten ein erhöhtes Risiko für Lichen simplex chronicus (RR 9,71), irritative Kontaktdermatitis (RR 9,60), Pruritus (RR 5,71), allergische Rhinitis (RR 1,78) und allergisches Asthma (RR 1,49).

**Schlussfolgerungen:**

Trotz unterschiedlicher methodischer Ansätze, die zu Abweichungen führten, kann die Prävalenz von NE mit etwa 0,26% angenommen werden. Überschneidungen und Fehlcodierungen sind mit anderen Formen von Ekzemen möglich, was die Notwendigkeit fundierter Diagnostik und standardisierter Kodierung nahelegt.

## EINLEITUNG

Das nummuläre Ekzem (NE) ist eine meist chronische, rezidivierende und juckende ekzematöse Hauterkrankung, die sowohl bei Kindern als auch bei Erwachsenen auftritt. Es unterscheidet sich von anderen Ekzemtypen aufgrund seines phänotypischen Erscheinungsbildes in Korrelation mit histologischen Befunden. Es wird ein kodominantes Th2/Th17‐Immunantwortmuster beobachtet, das darauf hindeutet, dass NE und AD einen gemeinsamen Entzündungsweg haben.^1^ Klinisch ist es durch entzündliche runde oder ovale erythematöse und ekzematöse Plaques auf meist trockener Haut gekennzeichnet.^1^, ^2^ Typischerweise sind die Läsionen multipel und variieren in der Größe von etwa 1 cm bis 6 cm im Durchmesser. Obwohl es in der dermatologischen Routineversorgung häufig vorkommt, ist es eine wenig erforschte Erkrankung, zu der nur wenige Studien zur Epidemiologie vorliegen.

Es wurde nachgewiesen, dass NE bei 13,5% der Personen mit atopischer Dermatitis,^3^ bei 3,5% aller dermatologischen Erkrankungen beziehungsweise ekzematösen Dermatitiden^4^, ^5^ oder bei 7% der Personen mit Handekzemen^6^ auftritt. In Deutschland wurden bislang keine epidemiologischen Daten veröffentlicht. Darüber hinaus gibt es weltweit nur wenige veröffentlichte Arbeiten zur Prävalenz und Inzidenz von NE. Studien zeigen, dass die Prävalenz von NE mit zunehmendem Alter steigt.^5^, ^7^, ^8^ Männer sind vorwiegend im Alter zwischen 50 und 65 Jahren betroffen, während Frauen insbesondere im Alter zwischen 15 und 25 Jahren betroffen sind. Die Diagnose wird hauptsächlich klinisch anhand der typischen scheibenförmigen Läsionen gestellt. Das histopathologische Erscheinungsbild ist für diese Art von Ekzem nicht spezifisch. Daher sollten andere klinisch ähnliche Hauterkrankungen ausgeschlossen werden.^9^ Überlappungen mit anderen dermatologischen Erkrankungen, darunter Kontaktdermatitis und atopische Dermatitis, wurden berichtet.^7^, ^10^

Angesichts der begrenzten Literatur sind für das Verständnis der Krankheitslast von NE, insbesondere für eine angemessene Gesundheitsplanung aktuelle bevölkerungsbasierte Daten von großer Bedeutung. Die vorliegende Studie schließt diese wissenschaftliche Lücke, indem sie einen umfassenden und longitudinalen Einblick in die Epidemiologie von NE in Deutschland liefert, basierend auf einer großen, nicht selektierten Population aus einer bundesweiten gesetzlichen Krankenversicherung. Ein zweites Ziel dieser Studie auf Basis von Routinedaten war die Entwicklung eines Konzepts zur internen Validierung der Daten als Grundlage für eine valide Schätzung der Prävalenz, Inzidenz und Schwere der NE in Deutschland.

## METHODIK

### Studiendesign und Datenquelle

Die gesetzliche Krankenversicherung (GKV) ist ein wesentlicher Bestandteil des deutschen Gesundheitssystems: Rund 89% der deutschen Bevölkerung (etwa 72 Millionen Menschen) sind bei einer der 95 gesetzlichen Krankenkassen versichert. Die restlichen 11% sind privat versichert. Die DAK‐Gesundheit (DAK‐G) ist eine große, bundesweit tätige Krankenkasse mit 5,5 Millionen Mitgliedern im Jahr 2022. Die in diesem Projekt analysierte Studienpopulation der DAK‐G ist eine anonymisierte 40%‐Zufallsstichprobe aller Versicherten, die zwischen *(1)* 2016 und 2020 und *(2)* 2018 und 2022 mindestens einen Tag versichert waren (54,9% Frauen, Durchschnittsalter 46,5 Jahre im Jahr 2022). Die Rechtsgrundlage und der Datenschutz der Stichprobe unterliegen dem Sozialgesetzbuch (SGB) und dem Bundesdatenschutzgesetz (BDSG). Die Daten der DAK‐G haben sich nach Bereinigung um Alter und Geschlecht als repräsentativ für die gesamte deutsche Bevölkerung erwiesen.^11^

Die GKV‐Daten enthalten alle abrechnungsrelevanten Informationen aus dem ambulanten und stationären Bereich, einschließlich Arbeitsunfähigkeit und aller verschreibungspflichtigen Medikamente im ambulanten Bereich sowie aller ambulanten Kontakte zu Ärzten, codierten Diagnosen, abgerechneten Leistungen und der zeitlichen Spezifizierung des Arztbesuchs auf Quartalsbasis.^12^ Insgesamt spiegeln die GKV‐Daten die Behandlungsprävalenz einer bestimmten Krankheit wider, wobei Personen, die nicht im Rahmen des GKV‐Systems behandelt werden, nicht erfasst werden.

### Falldefinition und Kovariaten

Für die interne Validierung wurden die Prävalenz und Inzidenz von NE anhand unterschiedlicher Falldefinitionen erfasst. Die Identifizierung von Personen mit NE erfolgte anhand der amtlichen Klassifikation zur Verschlüsselung von Diagnosen in Deutschland (ICD‐10‐GM‐Diagnose [L30.0]) während der ambulanten Versorgung oder des stationären Aufenthalts. Für die Inzidenz wurden unterschiedliche diagnosefreie Zeiträume (Wash‐out‐Perioden) definiert (Tabelle 1). Die Studienpopulation musste während der Beobachtungsjahre durchgehend versichert sein (mindestens ein Tag pro Quartal). Personen, die während des Beobachtungszeitraums verstorben sind, wurden nicht aus den Analysen ausgeschlossen.

**TABELLE 1:** Falldefinitionen zur Bestimmung der Prävalenz und Inzidenz des nummulären Ekzems (NE; ICD‐10‐GM L30.0) in deutschen Abrechnungsdaten der gesetzlichen Krankenversicherung.

	Falldefinition (Prävalenz)			Falldefinition (Inzidenz)		
Kriterien	A (≥ 1 NE dx) Basis‐Falldefinition	B (≥ 2 NE dx innerhalb eines Jahres)	C (≥ 2 NE dx innerhalb von drei Jahren)	I (2 Jahre dx‐frei)	II (3 Jahre dx‐frei)	III (4 Jahre dx‐frei)
(≥ 1 stationäre Haupt‐ oder Nebendiagnose NE)	**+**	**+**	**+**	**+**	**+**	**+**
***ODER***						
≥ 1 gesicherte ambulante Diagnose	**+**			**+**	**+**	**+**
≥ 1 gesicherte ambulante Diagnose in mindestens 2 Quartalen		**+**				
≥ 1 gesicherte ambulante Diagnose in mindestens 1 Quartal des Beobachtungsjahres und mindestens 1 in den beiden vorangegangenen Jahren)			**+**			
***UND***						
dx‐freie Zeit (ambulant und stationär) in den letzten 8 Quartalen				**+**		
dx‐freie Zeit (ambulant und stationär) für die letzten 12 Quartale					**+**	
dx‐freie Zeit (ambulant und stationär) in den letzten 16 Quartalen						**+**

*Abk*.: + in der Falldefinition enthalten; dx = Diagnose

Darüber hinaus wurde das gleichzeitige Auftreten von atopischer Dermatitis (AD) (ICD‐10‐GM L20) mit NE untersucht. Um den Anteil von Personen mit NE zu bestimmen, bei denen AD diagnostiziert wurde, wurde ein Sensitivitätstest durchgeführt. Zu diesem Zweck wurden Personen mit AD berücksichtigt, die im Prävalenzjahr keine NE aufwiesen, bei denen jedoch in den 2 Jahren vor und in den 2 Jahren nach dem Prävalenzjahr eine NE diagnostiziert wurde. Die folgenden ICD‐10‐GM‐Codes wurden analysiert, um andere begleitende Haut‐ und atopische Erkrankungen bei Personen mit NE im Vergleich zu Personen ohne NE zu schätzen (Tabelle S1 im Online‐Supplement).

Da GKV‐Routinedaten keine ausreichenden klinischen Informationen zur Charakterisierung des Schweregrads der Erkrankung liefern, werden Surrogatmarker betrachtet. Der Schweregrad der NE wurde somit anhand mindestens eines der folgenden Kriterien operationalisiert: *(1)* stationären Krankenhausaufenthaltes (NE Hauptdiagnose), *(2)* Arbeitsunfähigkeitstage, *(3)* Verordnungen systemischer Medikamente (Tabelle S2 im Online‐Supplement). Systemische Antibiotika und Antihistaminika wurden hierbei ausgeschlossen, da sie häufig zur Behandlung einer Reihe weiterer Erkrankungen eingesetzt werden und daher weniger krankheitsspezifisch sind.

### Statistische Analyse

Die administrativen Schätzungen der jährlichen Prävalenz‐ und Inzidenzraten werden als Prozentsätze mit den entsprechenden 95%‐Konfidenzintervallen (KI) für die Beobachtungsjahre 2016 bis 2022 angegeben. Alle Ergebnisse wurden nach Alter und Geschlecht für die deutsche Bevölkerung zum 31. Dezember des jeweiligen Jahres gemäß dem deutschen Statistischen Bundesamt (Destatis) direkt standardisiert. Die Raten wurden nach Alter, Geschlecht und regionaler Verteilung (Bundesland) stratifiziert. Das Alter wurde in zehn Altersgruppen und weitere Untergruppen (Entwicklungsphase in Jahren: 0 ≤ 6, 6 ≤ 12, 12 ≤ 18, 18 ≤ 25, 25 ≤ 50, > 50 und bis 18 Jahre) unterteilt. Zur Beschreibung der Daten kamen deskriptive statistische Verfahren zur Anwendung. Unterschiede in den Begleiterkrankungen zwischen den betrachteten Populationen (NE vs. ohne NE) wurden anhand der *Rate Ratio* (RR) mit den jeweiligen 95%‐Konfidenzintervallen (KI) dargestellt. Alle Analysen wurden mit dem Softwarepaket SAS for Windows^®^, Version 9.5 (SAS Institute Inc., Cary, North Carolina, USA) durchgeführt.

## ERGEBNISSE

Im Jahr 2022 wiesen 6431 der 2 366 437 Versicherten eine NE auf, was einer standardisierten Rate von 0,26% entspricht (Basis‐Falldefinition A, ≥ 1 NE‐Diagnose, Tabelle 2). Hochgerechnet auf die deutsche Bevölkerung würde dies einer geschätzten Zahl von 220 988 Personen entsprechen, bei denen eine NE diagnostiziert wurde. Das Durchschnittsalter der Personen mit NE lag bei 47,5 Jahren (Standardabweichung [SD] 25,3, Median 50), 57,4% waren Männer. Bei konservativeren Falldefinitionen lag die Prävalenzrate mit 0,07% (Falldefinition B, ≥ 2 NE‐Diagnosen innerhalb eines Jahres) und 0,11% (Falldefinition C, ≥ 2 NE‐Diagnosen innerhalb von 3 Jahren) deutlich niedriger. Im konservativsten Szenario würde die Gesamtzahl der Personen mit NE in Deutschland bei etwa 56 000 liegen. Die Prävalenzraten zeigten von 2016 bis 2022 einen leichten Rückgang, mit Ausnahme der Falldefinitionen B und C (≥ 2 NE‐Diagnosen).

**TABELLE 2:** Standardisierte Prävalenz des nummulären Ekzems nach verschiedenen Falldefinitionen innerhalb der Beobachtungsjahre 2016 bis 2022.

Jahr	N	Falldefinition A (Basis) (≥ 1 NE dx)				Falldefinition B (≥ 2 NE dx innerhalb 1 Jahres)				Falldefinition C (≥ 2 NE dx innerhalb von 3 Jahren)			
		*n*	*Rate,%*	*95%‐KI*	*Hochgerechnet auf die deutsche Bevölkerung*	*n*	*Rate,%*	*95%‐KI*	*Hochgerechnet auf die deutsche Bevölkerung*	*n*	*Rate,%*	*95%‐KI*	*Hochgerechnet auf die deutsche Bevölkerung*
2016	2 331 615	7643	0,30	0,30–0,30	250 400	1959	0,07	0,07–0,07	61 186	‐	‐		‐
2017	2 306 774	7487	0,30	0,30–0,31	252 285	1896	0,07	0,07–0,07	61 589	‐	‐		‐
2018	2 276 281	7189	0,29	0,29–0,30	244 655	1895	0,08	0,07–0,08	62 566	1 949	0,08	0,08–0,08	64 247
2019	2 246 743	7089	0,30	0,29–0,30	246 175	1865	0,07	0,07–0,08	62 082	2 822	0,11	0,11–0,11	93 741
2020	2 232 255	6544	0,27	0,27–0,27	226 987	1727	0,07	0,07–0,07	58 811	2 858	0,12	0,12–0,12	96 587
2021	2 336 344	6829	0,28	0,28–0,28	234 990	1805	0,07	0,07–0,07	61 042	2 938	0,12	0,12–0,12	100 075
2022	2 366 437	6431	0,26	0,26–0,26	220 988	1689	0,07	0,07–0,07	56 984	2 779	0,11	0,11–0,11	93 283

*Abk*.: dx, Diagnose; KI, Konfidenzintervall

Die Schätzung der Inzidenz änderte sich nur geringfügig bei Verlängerung der Wash‐out‐Periode – von 0,17% gemäß Basisfalldefinition I (2 Jahre diagnosefrei) auf 0,16% gemäß Falldefinition II und III (3 bzw. 4 Jahre diagnosefrei) (Tabelle 3). Darüber hinaus wurde gemäß den Falldefinitionen I und II (2 Jahre und 3 Jahre diagnosefrei) ein leichter Rückgang der Inzidenz beobachtet, während die Inzidenz gemäß Falldefinition III (4 Jahre diagnosefrei) in den beobachteten Jahren konstant bei 0,16% lag.

**TABELLE 3:** Standardisierte Inzidenz des nummulären Ekzems nach verschiedenen Falldefinitionen der Inzidenzfälle mit und ohne begleitende atopische Dermatitis innerhalb der Beobachtungsjahre 2018 bis 2022.

Jahr	Inzidenz Falldefinition I (Basis) (2 Jahre dx‐frei)					Inzidenz Falldefinition II (3 Jahre dx‐frei)					Inzidenz Falldefinition III (4 Jahre dx‐frei)				
	N	n	Rate,%	95%‐KI	Hochgerechnet auf die deutsche Bevölkerung	N	n	Rate,%	95%‐KI	Hochgerechnet auf die deutsche Bevölkerung	N	n	Rate,%	95%‐KI	Hochgerechnet auf die deutsche Bevölkerung
2018	2 135 290	4571	0,20	0,20–0,20	163 373	–	–	–		–	–	–	–		–
2019	2 120 101	4574	0,20	0,20–0,20	166 680	2 052 159	4253	0,19	0,19–0,19	157 969	–	–	–		–
2020	2 108 558	4113	0,18	0,18–0,18	149 102	2 048 737	3816	0,17	0,17–0,17	140 073	1 983 698	3614	0,16	0,16–0,16	135 569
2021	2 107 591	4124	0,18	0,18–0,18	152 877	2 040 477	3835	0,17	0,17–0,18	145 233	–	–	–		–
2022	2 138 829	3881	0,17	0,17–0,17	144 138	2 034 954	3573	0,16	0,16–0,16	137 182	1 971 391	3356	0,16	0,15–0,16	131 189

*Abk*.: dx, Diagnose; KI, Konfidenzintervall

**ABBILDUNG 1:**
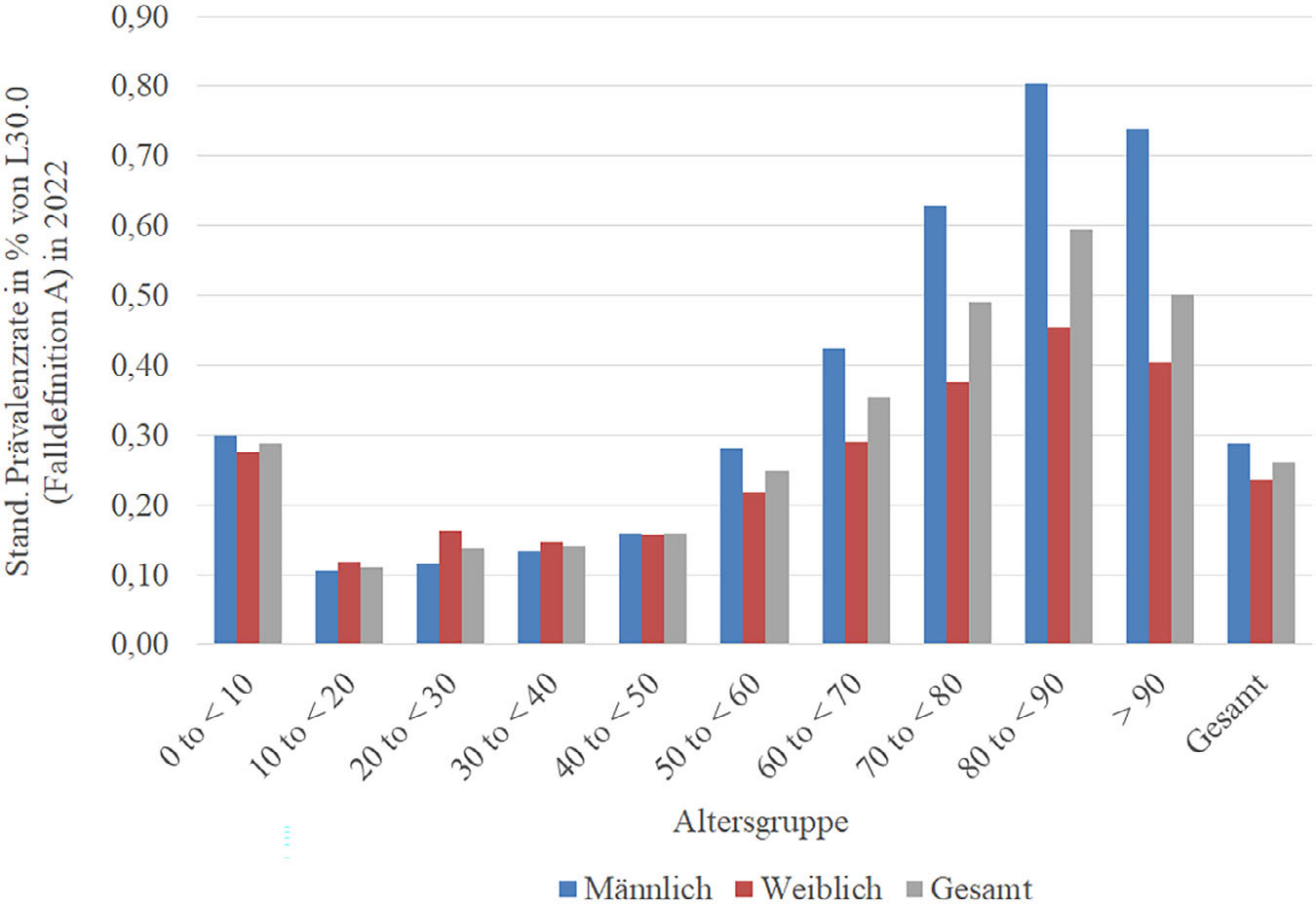
Standardisierte Prävalenz (nach Falldefinition A [≥ 1 Diagnose des NE]) nach Alter und Geschlecht im Jahr 2022 in Prozent.

Die Prävalenz und Inzidenz von NE steigt mit zunehmendem Alter, wobei Männer, insbesondere in der älteren Altersgruppe, häufiger betroffen sind (Abbildung 1 und 2). Höhere Raten wurden in der älteren Altersgruppe beobachtet, in der Männer mit NE einen ausgeprägten Aufwärtstrend zeigten und eine standardisierte Prävalenzrate von 0,80% und eine Inzidenzrate von 0,37% erreichten. Im Gegensatz dazu lag die Prävalenzrate für Frauen mit NE bei 0,45%. Die Alters‐ und Geschlechtsverteilung war über die verschiedenen Falldefinitionen hinweg konsistent und zeigte im Zeitverlauf keine erkennbaren Veränderungen.

Eine weitere Unterteilung in Altersklassen nach Entwicklungsphase zeigt, dass Kinder unter 6 Jahren (Abbildungen 4) und insbesondere unter 2 Jahren (Abbildung 3) besonders häufig von NE betroffen sind.

**ABBILDUNG 2:**
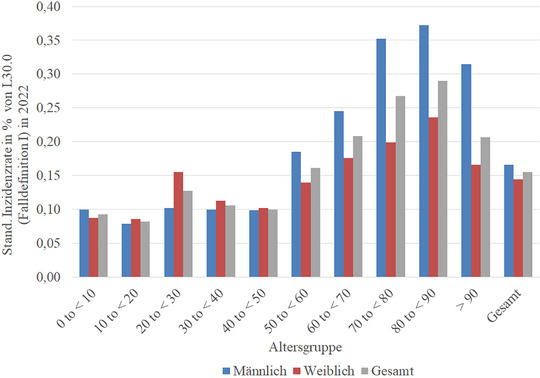
Inzidenzraten (nach Falldefinition I [diagnosefreier Zeitraum [ambulant und stationär] über 16 Quartale]) des nummulären Ekzems nach Alter und Geschlecht im Jahr 2022 in Prozent.

**ABBILDUNG 4:**
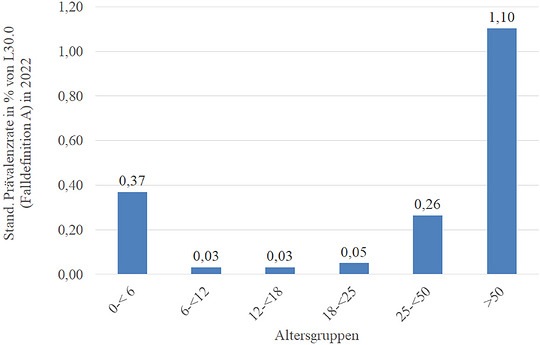
Standardisierte Prävalenz (%) des nummulären Ekzems bei Personen bis 18 Jahre im Jahr 2022 (Falldefinition A [≥ 1 Diagnose von NE]).

**ABBILDUNG 3:**
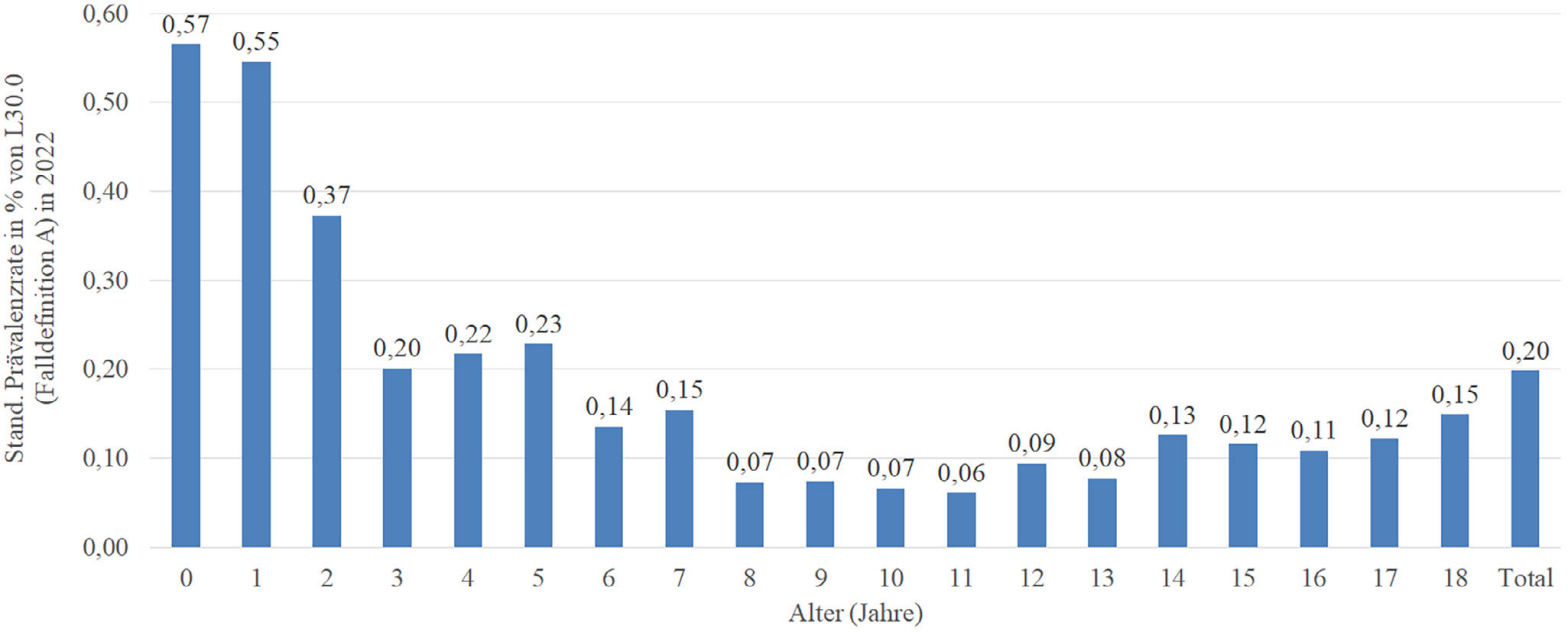
Standardisierte Prävalenz des nummulären Ekzems (Falldefinition A [≥ 1 Diagnose von NE]) nach Altersgruppen (Entwicklungsphase) im Jahr 2022 in Prozent.

**ABBILDUNG 5:**
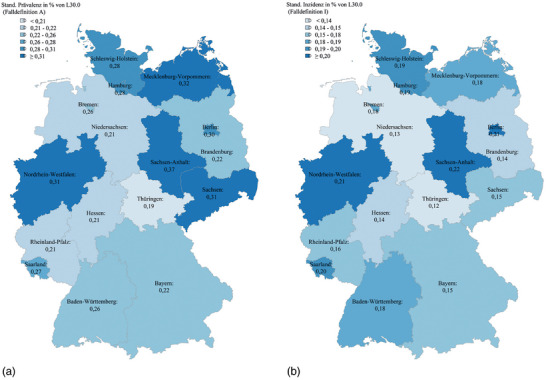
Standardisierte Prävalenz: (a) Falldefinition A (≥ 1 Diagnose des NE) und (b) Inzidenz: Falldefinition I (4 Jahre diagnosefrei) des NE im Jahr 2022 nach Bundesländern in Deutschland.

In Deutschland wurden deutliche regionale Unterschiede in der Prävalenz und Inzidenz von NE beobachtet. Im Jahr 2022 wurden die höchsten Prävalenz‐ und Inzidenzraten in den Bundesländern Sachsen‐Anhalt und Nordrhein‐Westfalen verzeichnet. Die niedrigsten Raten wurden hingegen in Thüringen festgestellt (Abbildung 5).

Darüber hinaus erhielten 18,0% der Personen mit einer NE im selben Jahr auch eine AD‐Diagnose (gemäß Basisfalldefinition A, ≥ 1 NE‐Diagnose). Ein Vergleich der Alters‐ und Geschlechtsverteilung von Personen, bei denen eine NE und eine AD diagnostiziert wurde oder die keine AD hatten, zeigt, dass eine AD‐Begleitdiagnose insbesondere im Kindesalter (0–10 Jahre) gestellt wurde (Abbildung S1 im Online‐Supplement). Die Sensitivitätsanalyse ergab, dass von den Personen, bei denen eine AD diagnostiziert wurde (Prävalenz 4,05%, Abbildung S2 im Online‐Supplement), bei 1,76% 2 Jahre vor oder nach dem Prävalenzjahr eine NE diagnostiziert wurde. Die Prävalenz gleichzeitig kodierter Begleiterkrankungen zeigte, dass Menschen mit NE ein höheres Risiko hatten, zusätzlich unter Haut‐ und atopischer Erkrankungen zu leiden, als Menschen ohne NE. Beispielsweise hatten Personen mit NE ein höheres Risiko, an exfoliativer Dermatitis (RR 11,0, 95%‐KI 3,6–33,5), Lichen simplex chronicus (RR 9,7, 95%‐KI 8,5–11,2), irritativer Kontaktdermatitis (RR 9,6, 95%‐KI 7,4–12,4), nicht näher bezeichneter Kontaktdermatitis (RR 7,0, 95%‐KI 5,9–8,3), seborrhoischer Dermatitis (RR 6,0, 95%‐KI 5,4–6,6) und Pruritus (RR 5,7, 95%‐KI 5,2–6,2) als Menschen ohne NE (Tabelle S2 im Online‐Supplement).

Im Jahr 2022 wurden 14,8% der Versicherten mit NE gemäß Falldefinition A (≥ 1 NE‐Diagnose) als schwer erkrankt identifiziert (Tabelle S3 im Online‐Supplement). Der Prozentsatz war mit 15,3% gemäß Falldefinition C (≥ 2 NE‐Diagnosen innerhalb von 3 Jahren) etwas höher. Zwischen 92,1% und 97,4% der Personen, bei denen NE diagnostiziert wurde, erhielten systemische Medikamente. Die Hospitalisierungsrate lag unter 10%, während der Anteil der Personen, die aufgrund von NE arbeitsunfähig waren, unter 3% lag.

## DISKUSSION

Ziel der vorliegenden Analyse der GKV‐Routinedaten war es, erste belastbare epidemiologische Daten zur Prävalenz und Inzidenz des nummulären Ekzems (NE) in der deutschen Bevölkerung zu gewinnen. Der verwendete Diagnosecode (ICD‐10‐GM L30.0) ist spezifisch für das nummuläre Ekzem. Über die ICD‐10‐Kodierung hinaus war eine weitere Verifizierung der Diagnose NE, beispielsweise anhand der Medikation, nicht möglich, da derzeit keine spezifische Therapie für NE existiert, die zur Verbesserung der diagnostischen Validität beitragen könnte.^13^ Es wurden verschiedene Falldefinitionen untersucht, um die Sensitivität der primären Fallkriterien in diesen Krankenversicherungsdaten zu ermitteln. Darüber hinaus wurde das Vorliegen des Diagnosecodes in verschiedenen Zeiträumen bewertet, um auch die Robustheit der Daten zu überprüfen. Die Prävalenz der Erkrankung lag 2019 zwischen 0,11% und 0,30% (abhängig von der Falldefinition). Die Prävalenz nach der Basisfalldefinition A (≥ 1 NE‐Diagnose) war während der COVID‐Pandemie zwischen 2020 und 2023 etwas niedriger. Die Prävalenz von NE wird durch Falldefinitionen, die Patientenbesuche oder Verschreibungen in mehr als einem Quartal berücksichtigen, unterschätzt, da die Erkrankung häufig in Schüben auftritt^9^, ^14^ und bei einigen Personen nur eine episodische Behandlung erforderlich ist. Daher ist das Kriterium von mindestens zwei Diagnosen in zwei verschiedenen Quartalen eines Jahres (Falldefinition B, ≥ 2 NE‐Diagnosen) zu streng, und zwei Diagnosen innerhalb eines längeren Zeitraums von 3 Jahren (Falldefinition C, ≥ 2 NE‐Diagnosen innerhalb von 3 Jahren) für diese spezifische Indikation nicht ausreichend. Es kann daher postuliert werden, dass die wahrscheinlichste Prävalenz von NE in den deutschen Abrechnungsdaten im Jahr 2019 bei 0,30% liegt, was etwa 250 000 Betroffenen in Deutschland entspricht.

Das nummuläre Ekzem stellt eine Variante des Ekzems dar, die durch eine ko‐dominante Typ‐3‐Immunantwort gekennzeichnet ist und sich klinisch von der AD unterscheidet. In der Praxis können jedoch bei einigen Patienten überlappende Erkrankungen festgestellt werden.^9^, ^15^ In den aktuellen GKV‐Daten wurde nur bei einem kleineren Anteil (etwa 18%) der Personen mit NE auch eine AD kodiert. Ein vergleichbares Muster lässt sich bei Personen mit AD‐Diagnose beobachten. In dieser Kohorte wurde bei 1,76% der Personen innerhalb eines Zeitraums von 2 Jahren vor oder nach einer AD‐Diagnose NE diagnostiziert, verglichen mit 0,26% in der Allgemeinbevölkerung. Das Auftreten anderer Formen von Ekzemen und Dermatitis bei NE lag ebenfalls unter 10%. Da ein gewisser Grad an Untererfassung nicht ausgeschlossen werden kann, weil einige Personen mit NE möglicherweise als AD kodiert wurden, wurde auch das gleichzeitige Auftreten von NE und anderen atopischen Erkrankungen untersucht. Wie erwartet zeigte sich bei Personen mit NE im Vergleich zu Personen ohne NE eine höhere Prävalenz von allergischer Rhinitis und allergischem Asthma. Diese Assoziationen waren jedoch weniger ausgeprägt als jene zwischen AD und NE.

Eine sorgfältige Interpretation dieser Ergebnisse deutet darauf hin, dass anhand von Abrechnungsdaten zwischen AD und NE unterschieden werden kann, auch wenn Überschneidungen bestehen – wie es auch andere Quellen belegen.^3^, ^7^, ^10^ Insgesamt unterstreichen diese Daten, ebenso wie andere Veröffentlichungen, den großen Bedarf an einer präziseren Definition des nummulären Ekzems sowohl auf klinischer als auch auf molekularer Ebene, um insbesondere Nicht‐Dermatologen eine genauere klinische Beurteilung zu ermöglichen. Es sind jedoch weitere Primärstudien unter Einbeziehung klinischer Daten erforderlich, um eine klarere Unterscheidung zwischen NE und AD sowie anderen ekzematösen Erkrankungen zu ermöglichen.

Die Ergebnisse bestätigen zudem eine hohe Prävalenz des NE bei jüngeren Kindern, die insbesondere mit einer AD verwechselt werden kann. Darüber hinaus zeigte sich bei Erwachsenen eine mit dem Alter zunehmende Prävalenz von NE, wie auch in der Literatur beschrieben.^5^ Männer sind in einem höheren Alter betroffen, typischerweise ab 50 Jahren, während Frauen etwas häufiger in einem jüngeren Alter zwischen 10 und 40 Jahren betroffen sind, wie eine US‐amerikanische Studie zuvor ebenfalls gezeigt hat.^7^ Zusätzlich zu den altersbedingten Unterschieden wurden regionale Unterschiede beobachtet, wie beispielsweise die hohe Prävalenz in Sachsen‐Anhalt und die unerwartet niedrige Prävalenz in Thüringen. Frühere Analysen haben eine insgesamt höhere Morbiditätslast in den östlichen Bundesländern gezeigt.^16^ Aus diesem Grund ist die vergleichsweise geringe Prävalenz von NE in Thüringen besonders auffällig und sollte weiter untersucht werden.

### Stärken und Schwächen

Die grundlegende Stärke dieser Analyse liegt in der großen Anzahl von Personen in den GKV‐Daten und der hohen Bevölkerungsabdeckung, da etwa 90% der deutschen Bevölkerung gesetzlich krankenversichert sind. Bei der Interpretation der Ergebnisse sind jedoch einige Einschränkungen zu berücksichtigen.

Die Populationen der verschiedenen Krankenkassen weisen erhebliche Unterschiede auf.^17^ Um diese Diskrepanzen zu verringern, wurden die Prävalenz‐ und Inzidenzraten nach Alter, Geschlecht und Bundesland auf die deutsche Bevölkerung standardisiert. Hinsichtlich der externen Validität hat eine Studie zu einer anderen Hauterkrankung (Psoriasis) gezeigt, dass die epidemiologischen Ergebnisse der DAK‐G‐Daten ohne Einschränkung auf die GKV‐Bevölkerung übertragen werden können, sofern sie standardisiert sind.^11^ Es ist jedoch zu beachten, dass die Ergebnisse in ihrer Verallgemeinerbarkeit auf die Gesamtbevölkerung eingeschränkt sein können, da die Routinedaten privat versicherte oder nicht versicherte Personen nicht erfassen.

Im Allgemeinen werden Routinedaten zu Abrechnungszwecken und nicht zu Forschungszwecken erhoben. Daher enthalten die Daten nur Leistungen, die vom gesetzlichen Krankenversicherungssystem erstattet werden. Folglich liegen keine Informationen über Gesundheitsleistungen vor, die von den Versicherten selbst oder von Dritten bezahlt werden. Analysen von Krankenversicherungsdaten unterschätzen möglicherweise die tatsächliche Erkrankungshäufigkeit, da nicht alle Erkrankungen zu einer Inanspruchnahme des Gesundheitssystems führen. Darüber hinaus liefern die Daten keine Informationen darüber, ob und wann Menschen ihre verordneten Medikamente nach Erhalt in der Apotheke tatsächlich einnehmen. Dies kann auf eine Reihe von Faktoren zurückzuführen sein, darunter unzureichende oder unangemessene Differenzialdiagnosen, Fehlklassifikationen oder das Kodierungsverhalten des Arztes. Inwieweit der präzisen Kodierung von ekzematösen Erkrankungen in der Routineversorgung Aufmerksamkeit geschenkt wird, ist unklar, insbesondere angesichts der unterschiedlichen dermatologischen Ausbildung und Erfahrung. Im Fall des NE können die Unsicherheiten hinsichtlich seiner Ätiologie und Pathophysiologie in Verbindung mit dem Fehlen allgemein anerkannter Diagnosekriterien zu einer weiteren uneinheitlichen Kodierung beitragen. Darüber hinaus kann die Unterkodierung von NE zugunsten der AD auf die größere Anzahl von Behandlungen zurückgeführt werden, die unter der letzteren Diagnose zugelassen und erstattet sind. Dies kann zu einer systematischen Verzerrung der prävalenzbasierten Schätzungen führen.

Zudem enthalten die Routinedaten keine klinischen Informationen zum Schweregrad der Erkrankung, zur Lebensqualität oder detaillierte persönliche Informationen wie Gewicht, Lebensstil und Bildungsstand.^17^ Es ist wichtig zu erkennen, dass die zur Quantifizierung einer schweren Form verwendeten Ersatzparameter nur Annäherungswerte für das tatsächliche Ausmaß sind. Die Daten deuten darauf hin, dass der Schweregrad der Erkrankung möglicherweise etwas überschätzt wird, da die untersuchten Medikamente (zwischen 92% und 98% erhielten ein Medikament) auch bei anderen Hauterkrankungen wie AD eingesetzt werden.

### Fazit

Diese Studie unterstreicht die Herausforderungen bei der genauen Schätzung der Prävalenz und Inzidenz des NE aufgrund der Verwendung unterschiedlicher Falldefinitionen auf der Grundlage von Routinedaten. Darüber hinaus wird die Notwendigkeit einer harmonisierten Leitlinie hervorgehoben, welche die Qualität sowohl der Diagnose als auch der Behandlung von Personen mit NE verbessern und damit zu genaueren Prävalenz‐ und Inzidenz schätzungen führen könnte.

## DANKSAGUNG

Wir danken der DAK‐G für ihre Zusammenarbeit und die Bereitstellung der Daten. Darüber hinaus danken wir dem Scientific Communication Team des IVDP, insbesondere Paula Willer für die redaktionelle Bearbeitung.

Open access Veröffentlichung ermöglicht und organisiert durch Projekt DEAL.

## FINANZIERUNG

Das Projekt wurde finanziell von Almirall unterstützt. Es handelte sich um eine unabhängige, von Forschern initiierte Studie, die von Almirall unterstützt wurde. Almirall hatte keinen Einfluss auf die Konzeption, Analyse oder Interpretation der Ergebnisse dieser Studie.

## STANDARDS UND ETHIK

Die Studie wurde in Übereinstimmung mit den Grundsätzen der Deklaration von Helsinki durchgeführt. Wir haben die STROBE‐ und STROSA‐Erklärungen sowie die Kriterien einer nationalen Leitlinie für gute Praxis berücksichtigt.^18^ Gemäß der *Good Practice of Secondary Data Analysis*, einer nationalen Leitlinie für die Verwendung von Routinedaten, ist eine Genehmigung durch eine Ethikkommission nicht erforderlich.^19^, ^20^

## INTERESSENKONFLIKT

K.E. war als Berater und/oder bezahlter Sprecher für AbbVie, Almirall, Apogee, Boehringer Ingelheim, BMS, Janssen, LEO, Lilly, Novartis, Pfizer, Sanofi, Sitryx und UCB tätig. Er ist Anteilseigner und Mitgründer von Dermagnostix und Dermagnostix R&D. M.A. war als Berater, Dozent und/oder Forscher tätig und/oder hat institutionelle Forschungszuschüsse von Unternehmen erhalten, die Medikamente gegen nummuläres Ekzem herstellen, darunter AbbVie, Almirall, Beiersdorf, Eli Lilly, Galderma, LEO, Novartis, Pfizer und Sanofi. Alle übrigen Autoren erklären, dass kein Interessenkonflikt besteht.

## Supporting information



Supplementary information

Supplementary information

Supplementary information

Supplementary information

Supplementary information
