# Supplementary material for: Epidemiologie des nummulären Ekzems – methodische Ansätze und Ergebnisse aus bundesweiten Routinedaten
Source: J Dtsch Dermatol Ges. 2026 Jul 7;24(7):886–95. [Article in German] doi: 10.1111/ddg.15932_g (PMC13340976; doi:10.1111/ddg.15932_g)
Supplement: Supplementary file 1 — Supplementary information [file DDG-24-886-s004.docx]

Ergänzte Tabelle S1 Prävalenz von Hauterkrankungen und atopischen Erkrankungen, die gleichzeitig bei Menschen mit nummulären Ekzems (NE) (Basisdefinitionsfall A, ≥ 1 Diagnose von NE (N = 6.431)) im Vergleich zu Personen ohne NE (N = 2.369.694) im Jahr 2022 kodiert wurden

|  |  | **NE** | | **Ohne NE** | |  |
| --- | --- | --- | --- | --- | --- | --- |
|  | **ICD-10-GM** | **n** |  | **n** | **%** | **Rate Verhältnis (95 % KI)** |
| Exfoliative Dermatitis | L26 | 3 | 0,05 | 9 | 0,00 | 10,98 (3,60–33,48) |
| Lichen simplex chronicus | L28 | 195 | 3,03 | 7.431 | 0,31 | 9,71 (8,44–11,18) |
| Reizkontaktdermatitis | L24 | 5 | 0,87 | 2.117 | 0,09 | 9,60 (7,40–12,44) |
| Nicht näher bezeichnete Kontaktdermatitis | L25 | 134 | 2,08 | 7.098 | 0,30 | 6,97 (5,88–8,26) |
| Seborrhoische Dermatitis | L21 | 378 | 5,88 | 24.141 | 1,02 | 5,99 (5,40–6,64) |
| Pruritus | L29 | 588 | 9,14 | 40.595 | 1,71 | 5,71 (5,24–6,21) |
| Atopische Dermatitis | L20 | 1.155 | 17,96 | 93.997 | 3,97 | 5,25 (4,93–5,59) |
| Allergische Kontaktdermatitis | L23 | 398 | 6,19 | 31.259 | 1,32 | 4,89 (4,42–5,40) |
| Dermatitis aufgrund von innerlich eingenommenen Substanzen | L27 | 71 | 1,10 | 6.505 | 0,27 | 4,02 (3,19–5,08) |
| Alopecia areata | L63 | 45 | 0,85 | 5.280 | 0,22 | 3,22 (2,40 – 4,31) |
| Windeldermatitis | L2 | 6 | 0,95 | 10.027 | 0,42 | 2,25 (1,75–2,89) |
| Allergische Rhinokonjunktivitis | J30 | 891 | 13,8 | 195.810 | 8,26 | 1,78 (1,66–1,91) |
| Allergisches Asthma | J45 | 762 | 11,85 | 196.355 | 8,29 | 1,49 (1,38–1,60) |
