# Supplementary material for: Epidemiologie des nummulären Ekzems – methodische Ansätze und Ergebnisse aus bundesweiten Routinedaten
Source: J Dtsch Dermatol Ges. 2026 Jul 7;24(7):886–95. [Article in German] doi: 10.1111/ddg.15932_g (PMC13340976; doi:10.1111/ddg.15932_g)
Supplement: Supplementary file 2 — Supplementary information [file DDG-24-886-s001.docx]

Ergänzte Tabelle S2 Berücksichtigte systemische Arzneimittel in der NE- und AD-Behandlung

| **Arzneimittelgruppe** | **Wirkstoff** | **ATC** |
| --- | --- | --- |
| Systemische Biologika | Dupilumab | D11AH05 |
|  | Tralokinumab | D11AH07 |
| Januskinase-Inhibitoren | Baricitinib | L04AA37 (bis 2023) |
|  | Upadacitinib | L04AA44 (bis 2023) |
| Systemische Nicht-Biologika  (konventionell) | Methotrexat | L01BA01, L04AX03, M01CX01 |
|  | Mycophenolsäure | L04AA06 |
|  | Alitretinoin | D11AH04 |
|  | Azathioprin | L04AX01 |
|  | Ciclosporin | L04AD01 |
|  | Methoxsalen | D05BA02 |
|  | Trioxysalen | D05BA01 |
| Systemische Glukokortikosteroide (GCS) | Glukokortikosteroide | H02AB |
|  | - Betamethason-Depot | H02AB51 |
|  | Methylprednisolon-Depot | H02AB54 |
|  | - Prednisolon-Depot | H02AB56 |
|  | - Triamcinolon-Depot | H02AB58 |
