# Supplementary material for: Epidemiologie des nummulären Ekzems – methodische Ansätze und Ergebnisse aus bundesweiten Routinedaten
Source: J Dtsch Dermatol Ges. 2026 Jul 7;24(7):886–95. [Article in German] doi: 10.1111/ddg.15932_g (PMC13340976; doi:10.1111/ddg.15932_g)
Supplement: Supplementary file 3 — Supplementary information [file DDG-24-886-s005.docx]

Ergänzte Tabelle S3 Prävalenz eines schweren nummulären Ekzems (NE) gemäß den verschiedenen Falldefinitionen von 2016 bis 2022; „schweres NE“ wurde definiert als ≥ 1 Krankschreibung oder ≥ 1 Krankenhausaufenthalt oder ≥ 1 systemische Medikamentenverschreibung im Zusammenhang mit NE)

| **Jahr** | **Falldefinition** | **Versicherte Personen mit NE (N)** | **Schwere NE, n (%)** | **Systemische Medikamente, %** | **Krankschreibung, %** | **Krankenhausaufenthalt, %** |
| --- | --- | --- | --- | --- | --- | --- |
| 2016 | A, ≥ 1 NE | 7.643 | 1073 (14,04) | 94,22 | 2,33 | 5,68 |
|  | B, ≥ 2 NE innerhalb eines Jahres | 1.959 | 313 (15,98) | 94,25 | 1,60 | 6,71 |
| 2017 | A, ≥ 1 NE | 7.487 | 1057 (14,12) | 95,18 | 2,74 | 5,11 |
|  | B, ≥ 2 NE innerhalb eines Jahres | 1.896 | 295 (15,56) | 95,59 | 2,71 | 4,75 |
| 2018 | A, ≥ 1 NE | 7.189 | 998 (13,88) | 95,49 | 1,70 | 5,31 |
|  | B, ≥ 2 NE innerhalb eines Jahres | 1.895 | 290 (15,30) | 94,48 | 2,41 | 6,55 |
|  | C, ≥ 2 NE innerhalb von drei Jahren | 1.949 | 316 (16,21) | 92,09 | 2,85 | 9,81 |
| 2019 | A, ≥ 1 NE dx | 7.089 | 1038 (14,64) | 94,70 | 2,50 | 5,01 |
|  | B, ≥ 2 NE dx innerhalb eines Jahres | 1.865 | 327 (17,53) | 94,19 | 3,36 | 4,2 |
|  | C, ≥ 2 NE dx innerhalb von drei Jahren | 2.822 | 478 (16,94) | 93,51 | 2,51 | 6,28 |
| 2020 | A, ≥ 1 NE dx | 6.544 | 862 (13,17) | 96,40 | 1,86 | 3,95 |
|  | B, ≥ 2 NE dx innerhalb eines Jahres | 1.727 | 254 (14,71) | 95,28 | 2,37 | 4,35 |
|  | C, ≥ 2 NE dx innerhalb von drei Jahren | 2.858 | 435 (15,22) | 94,48 | 2,31 | 6,24 |
| 2021 | A, ≥ 1 NE dx | 6.829 | 938 (13,74) | 96,27 | 1,28 | 4,16 |
|  | B, ≥ 2 NE dx innerhalb eines Jahres | 1.805 | 298 (16,51) | 97,32 | 0,67 | 3,69 |
|  | C, ≥ 2 NE dx innerhalb von drei Jahren | 2.938 | 451 (15,35) | 95,79 | 0,89 | 5,54 |
| 2022 | A, ≥ 1 NE dx | 6.431 | 949 (14,76) | 97,37 | 1,48 | 3,48 |
|  | B, ≥ 2 NE dx innerhalb eines Jahres | 1.689 | 283 (16,76) | 98,23 | 1,41 | 3,89 |
|  | C, ≥ 2 NE dx innerhalb von drei Jahren | 2.779 | 424 (15,26) | 97,64 | 1,65 | 4,01 |
