# Supplementary figures and images for: Epidemiologie des nummulären Ekzems – methodische Ansätze und Ergebnisse aus bundesweiten Routinedaten
Source: J Dtsch Dermatol Ges. 2026 Jul 7;24(7):886–95. [Article in German] doi: 10.1111/ddg.15932_g (PMC13340976; doi:10.1111/ddg.15932_g)

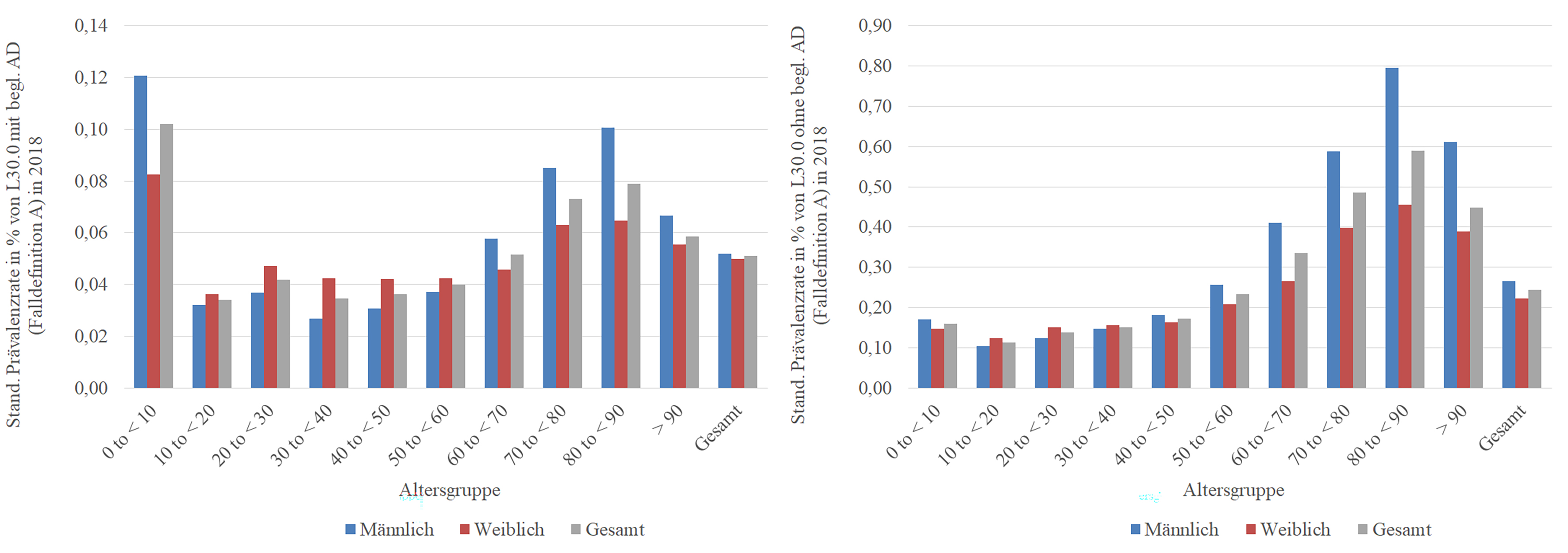

Supplement: Supplementary file 4 — Supplementary information [file DDG-24-886-s003.jpg]

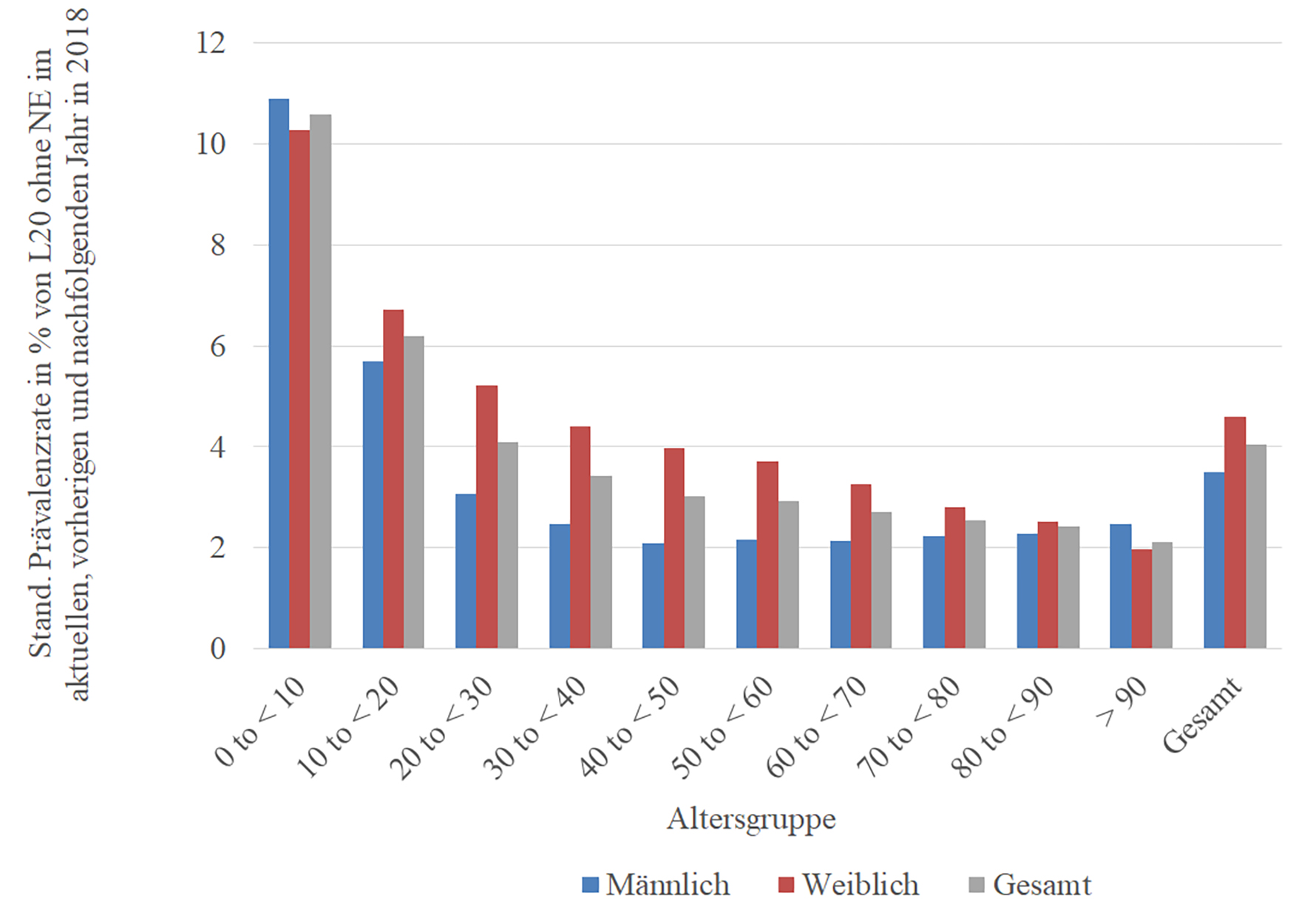

Supplement: Supplementary file 5 — Supplementary information [file DDG-24-886-s002.jpg]
